# A shared tissue transcriptome signature and pathways in psoriasis and ulcerative colitis

**DOI:** 10.1038/s41598-022-22465-w

**Published:** 2022-11-17

**Authors:** Li Xi, Sandra Garcet, Zhan Ye, Kenneth Hung, Mina Hassan-Zahraee, Elizabeth Kieras, James G. Krueger, Craig Hyde, Elena Peeva

**Affiliations:** 1 Pfizer Inc., Cambridge, MA USA; 2 Laboratory of Investigational Dermatology, The Rockefeller University, New York, NY 10065 USA

**Keywords:** Ulcerative colitis, Skin diseases

## Abstract

Despite multiple efficacious therapies in common between psoriasis (PS) and Ulcerative Colitis (UC), mechanisms underlying their common pathophysiology remain largely unclear. Here we sought to establish a link by evaluating expression differences and pathway alterations in diseased tissues. We identified two sets of differentially expressed genes (DEGs) between lesional and nonlesional tissues in meta-analyses of data collected from baseline samples in 3 UC and then 3 PS available clinical studies from Pfizer. A shared gene signature was defined by 190 DEGs common to both diseases. Commonly dysregulated pathways identified via enrichment analysis include interferon signaling, partly driven by genes IFI6, CXCL9, CXCL10 and CXCL11, which may attract chemotaxis of Th1 cells to inflammatory sites; IL-23 pathway (IL-23A, CCL20, PI3, CXCL1, LCN2); and Th17 pathway except IL-17A. Elevated expression of costimulatory molecules ICOS and CTLA4 suggests ongoing T-cell activation in both diseases. The clinical value of the shared signature is demonstrated by a gene set improvement score reflecting post-treatment molecular improvement for each disease. This is the first study using transcriptomic meta-analysis to define a tissue gene signature and pathways dysregulated in both PS and UC. These findings suggest immune mechanisms may initiate and sustain inflammation similarly in the two diseases.

## Introduction

Although many diseases affect different systems of the body, some are associated conditions with shared underlying inflammatory etiology^1^. PS and UC are both among the most common immune-mediated chronic relapsing diseases. The known association of PS with common gastrointestinal diseases suggest that PS is a red flag for gut inflammation in general, even if particularly UC and PS share the same pathogenetic mechanisms that account for the same response to drugs used in the 2 diseases^2,3^. In particular, studies have shown PS’s association with UC could possibly be as an independent concomitant immune-mediated diseases (IMID), or a manifestation of underlying UC, or a paradoxical adverse event of anti-tumor necrosis factor (TNF) and IL-17A inhibitor therapies^4,5^. Factors of the noted association could be related to shared genetic abnormalities, common cytokine-driven inflammation including interleukin IL-23 and Th17 pathways or environmental factors^6^. Nevertheless, the link between psoriasis and UC is currently far from clear^6^.

Psoriasis (PS) is a chronic inflammatory skin disease involving activated T cells, including helper type 17 (Th17) cells, keratinocytes, and dendritic cells^7^, while Ulcerative Colitis (UC) is a form of chronic inflammatory bowel disease (IBD) characterized by changes in the mucosal architecture and immune cell infiltration and an elevated concentration of inflammatory cytokines that influence the activity of T cells, including Th17 cells and Tregs, both of which are considered crucial within the pathogenic process^8^.

It is established that both innate and adaptive immunity are essential in initiating and maintaining chronic inflammation in UC, while the roles both these segments of the immune system played are also common to other immune-mediated diseases (IMIDs) including PS^6^.

Because of these shared features, several drugs are used to treat PS and UC. A comprehensive review of 132 clinical trials^9^ reported that Ustekinumab (a monoclonal antibody directed against interleukin IL-23 and IL-12), Infliximab and Adalimumab (TNFα inhibitors) have been effectively used in the treatment of both PS and UC. It is important to note roles of other inflammatory mediators such as IL-17A/F have shown to differ in the two diseases as reflected in response to IL-17 targeting therapies in PS and UC^10,11^. Anti-IL17 has been successful in PS, whereas not effective in UC or CD^10,11^.

Although there has been increasing interest in the role of IL-17 family in the development of autoimmune and inflammatory conditions, the understanding about IL-17 contribution in the pathogenesis of inflammatory bowel disease (IBD) is limited and inconsistent findings generate discussion in that field^12^. IL-17 has emerged as a surprising arbiter of intestinal homeostasis on the basis of clinical observations of exacerbated IBD symptoms in patients with psoriasis treated with biologics that inhibit the IL-17 pathway^13^.

Published studies have defined differentially expressed genes (DEGs) between lesional and nonlesional skin in PS patients^14–16^ and between lesional and nonlesional colon samples from UC patients^17,18^. However, none have compared transcriptional changes in PS skin and UC mucosal tissues from clinical studies and to investigate their shared pathogenesis mechanisms at molecular levels. These shared DEGs will provide biological insights on common mechanisms that may previously be unknown. These may have important implications for drug development, as these commonalities could represent key pathophysiologic pathways that drive the diseases, as opposed to others that might simply be epi-phenomenon.

The purpose of this study was to identify genes and pathways altered in both PS and UC pathogenesis in paired skin/colon tissue biopsies from patients. To establish a link between PS and UC at the transcriptional level, we performed two sets of meta-analyses of transcriptomic data of baseline samples from 6 Pfizer clinical studies, namely, 3 PS studies (skin biopsies) and 3 UC (colon biopsies). The samples analyzed in this study were baseline samples and patients did not receive any treatment when these samples were collected. We defined a shared signature in PS and UC that includes 190 DEGs covering a spectrum of innate and adaptive components of the immune system. Our findings highlight the important role of IL-23 pathway in promoting inflammation in both conditions; and Th17 and PPAR Signaling pathways in the physiopathology of the two diseases, the degree of involvement of each dysregulated pathway may be different between PS and UC, though. Of note, IL-17A is only present in PS transcriptome, while its signal is below limit of detection in UC datasets, hence is not part of the shared tissue signature.

## Results

### Intersection of two meta-analyses defined a shared gene signature between PS and UC representing core disease pathogenesis in tissues from both diseases

**Figure 1:**
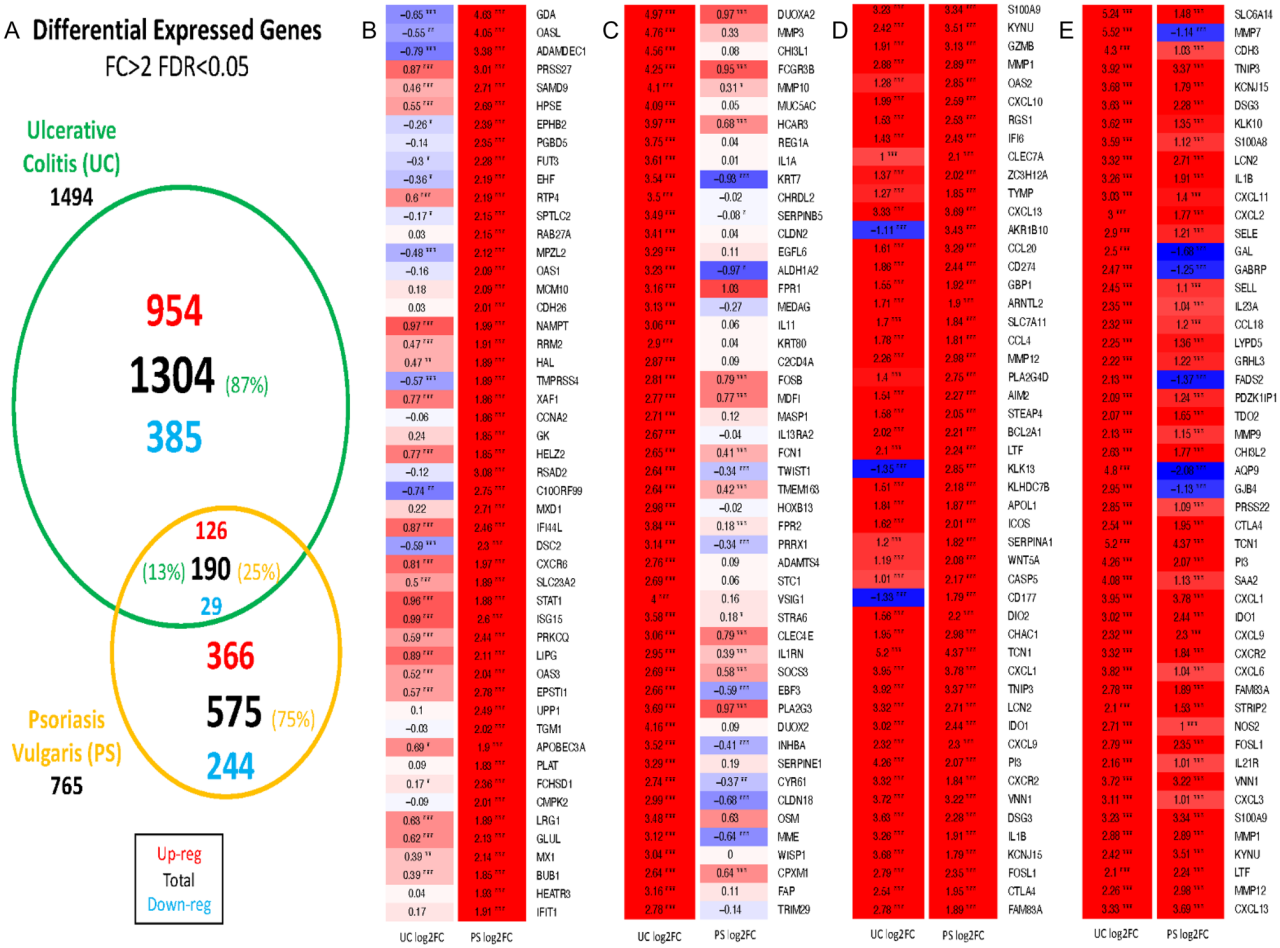
(**A**) Differential expressed genes FC > 2 FDR < 0.05. (**B**) Venn diagram of DEGs with |FC|> 2 and FDR < 0.05. UC lesional tissue vs. nonlesional tissue/PS lesional tissue vs. nonlesional tissue. (**B**) Top 50 upregulated PS DEGs among unique PS genes. (**C**) Top 50 upregulated UC DEGs among unique UC genes. (**D**) Top 50 upregulated PS DEGs among shared UC and PS genes. (**E**) Top 50 upregulated UC DEGs among shared UC and PS genes. There are 35 differentially expressed genes for both diseases but in different directions, what makes up/down numbers differ from the total number of DEGs.

To mitigate heterogeneity between studies, we considered the effect size of individual studies and penalized cross-study heterogeneity at the gene level. The meta-analysis identified 1080 upregulated and 410 downregulated genes in UC studies and 492 upregulated and 273 downregulated genes in PS studies. The differentially expressed genes (DEGs) in UC and PS, defined by fold change (FC) > 2 and FDR < 0.05, are presented in Fig. 1. A total of 190 genes were differentially expressed in paired lesional tissues compared to nonlesional tissues in both diseases, among which 126 have increased expression in lesional tissue, while 29 genes exhibited decreased expression (Fig. 1). These 190 genes were defined as the shared PS and UC signature.

Among shared genes, some general patterns were observed: Interferon Signaling enrichment is suggested by the DEGs CXCL9, CXCL10, and CXCL11. These chemokines may chemoattract Th1 T cells to inflammatory sites^19^. The chemokine CCL20 is also upregulated in both diseases, and this chemokine may increase the trafficking of immature CD11c + dendritic cells, as well as Th17 T cells^19^ into inflammatory sites. T-cell-associated genes (CD274, CD28, and GZMB) are overexpressed in both diseases. IL-1b’s over-expression in both diseases could lead to increased neutrophil presence through the downstream production of CXCL chemokines^20,21^ as evidenced by increased CXCR2 and LCN2 expression in both diseases.

Of the 35 genes with significant changes in different directions in PS and UC, 23 were under-expressed in PS lesional skin and over-expressed in UC colon tissues. Majority of these genes are related to metabolic pathways. FADS2, part of the fatty acid metabolism pathway, was downregulated in PS, while upregulated in UC. Another gene with opposite expression patterns, AQP9, has been reported to play a role in regulation of physiological properties in tight junctions in UC^22^; it exhibits lower expression in psoriatic lesions and higher expression in UC. Among the other 12 genes upregulated in PS and downregulated in UC is the neutrophil activation marker CD177, which modulates neutrophil migration through activation-mediated integrin and chemoreceptor regulation^23^. The other 20 DEGs with opposite changes in two diseases warrant further investigation.

We further examined the sets of DEGs unique to each disease, that is, not among the 190 DEGs shared by both diseases (Fig. 1A). The top 50 DEGs detected in PS only were compared to the top 50 genes detected in UC only (Fig. 1B vs. C). In the group of 190 shared DEGs, expression was ordered by highest expression in PS (Fig. 1D) or highest expression in UC (Fig. 1E). Unique PS genes include STAT1 and several Interferon-associated gene products (OASL, OAS1, IFI44L, ISG15, OAS3, MX1, and IFIT1)^24^, suggesting stronger interferon signaling. Genes with elevated expression in UC include some proteases (MMP-3, MMP-10) and inflammatory cytokines (IL-1A, IL-11, OSM), potentially suggesting their roles in regulation of epithelial barrier function, immune response, fibrosis and wound healing^3^.

Identified in our meta-analyzed datasets (not shown in figures), IL-17A/F/C all exhibited increased expression in baseline psoriatic lesional skin, paralleled by a significant decrease in IL-17RA/B/C/D/E. In UC baseline colon lesional tissues, only IL-17REL (Genome-wide association study identifies IL-17REL as a risk locus for UC)^25^ was significantly upregulated, while IL-17RB was significantly downregulated. IL-17A and IL-17F levels were below the limit of detection in colon tissues in the datasets used in the meta-analysis in the current study. Therefore, IL-17A and IL-17F are not part of the shared gene signature. This may or may not partially explain that IL-17A inhibitors have demonstrated effective therapeutic benefits in PS but not in UC^26^.

### Pathway analysis highlights a strong enrichment of IL-17 signaling and PPAR signaling in PS and UC

**Figure 2:**
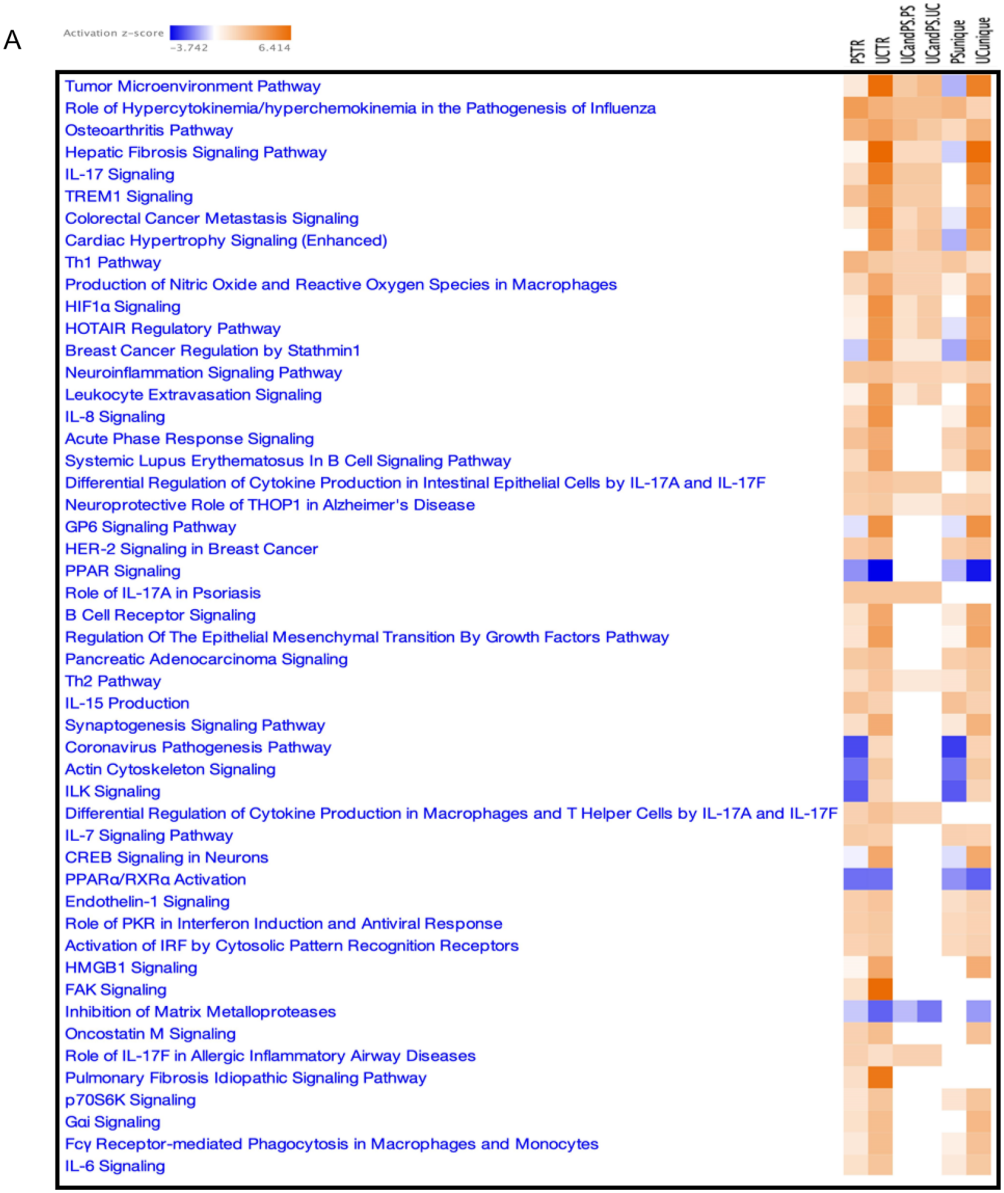

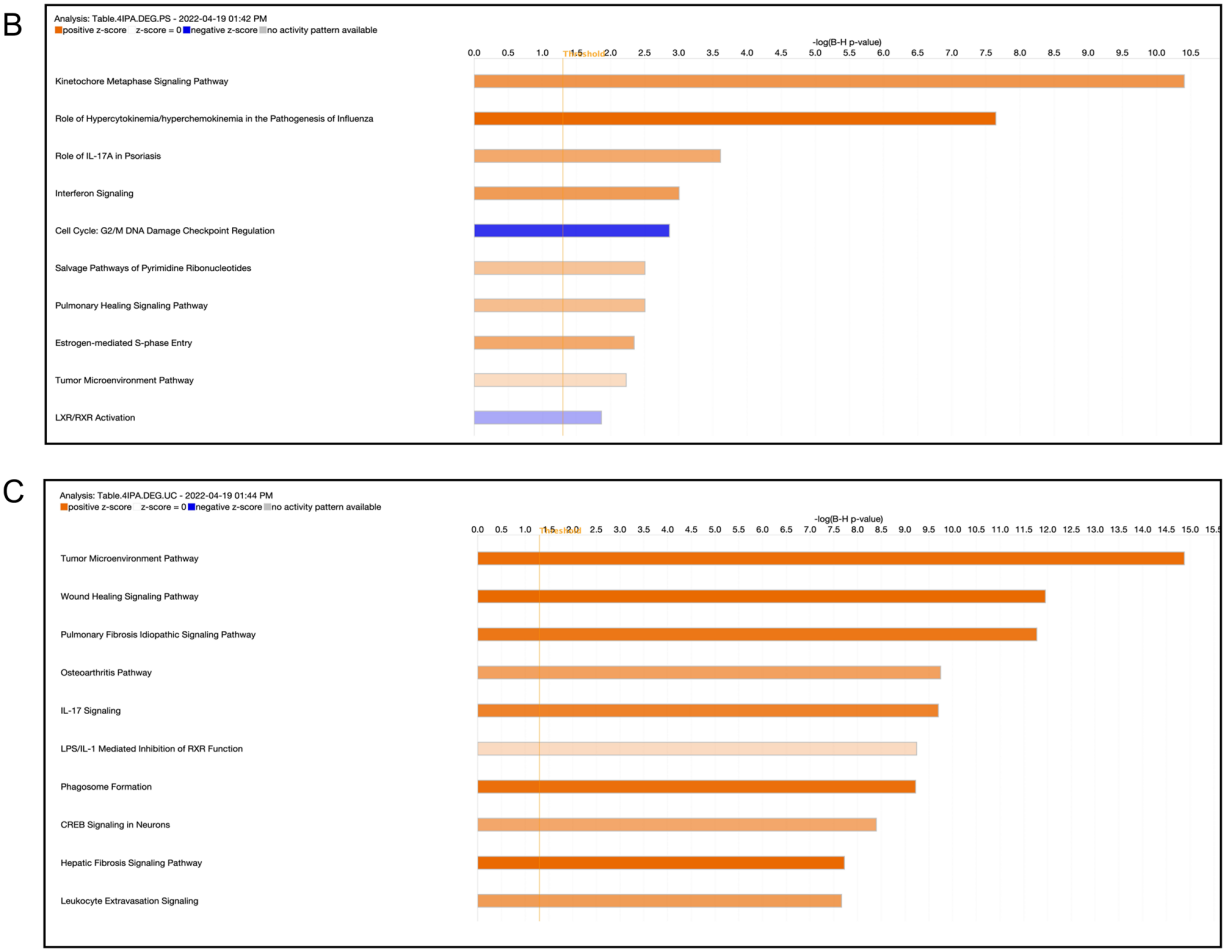
Enriched Canonical Pathways identified via IPA. (**A**) Top pathways enriched in the follow: (a) PSTR: PS transcriptome, (b) UCTR: UC transcriptome, (c) UC and PS.PS: zscore in PS for DEGs common in UC and PS, (d) UC and PS.UC: zscore in UC for DEGs common in UC and PS, (e) PS.unique: present only in PS, (f) UC.unique: present only in UC. Heatmap of z-scores for most significant canonical pathways were generated through the use of IPA (QIAGEN Inc., https://www.qiagenbioinformatics.com/products/ingenuity-pathway-analysis). Z-scores are based on the number of differentially expressed genes that map to every pathway at each subset; adjusted p-values from Fisher’s exact test have been used for significance criteria. (**B**) Top 10 pathways enriched in PS. Barplot of adjusted p-values for top 10 significant Canonical Pathways in PS pathways were generated through the use of IPA (QIAGEN Inc., https://www.qiagenbioinformatics.com/products/ingenuity-pathway-analysis). Z-scores, also generated through the use of IPA (QIAGEN Inc., https://www.qiagenbioinformatics.com/products/ingenuity-pathway-analysis), are based on the number of differentially expressed genes that map to every pathway at each subset; adjusted p-values from Fisher’s exact test have been used for significance criteria. (**C**) Top 10 pathways enriched in UC. Barplot of adjusted p-values for top 10 significant Canonical Pathways in PS pathways were generated through the use of IPA (QIAGEN Inc., https://www.qiagenbioinformatics.com/products/ingenuity-pathway-analysis). Z-scores, also generated through the use of IPA (QIAGEN Inc., https://www.qiagenbioinformatics.com/products/ingenuity-pathway-analysis), are based on the number of differentially expressed genes that map to every pathway at each subset; adjusted p-values from Fisher’s exact test have been used for significance criteria.

To dissect and compare pathways represented in PS or UC, enrichment analysis was performed using Ingenuity Pathway Analysis (IPA, QIAGEN) on disease related DEGs, including full DEG sets for both diseases (Fig. 2Aa,b), shared genes between the diseases (Fig. 2Ac,d), and unique genes for each disease (Fig. 2Ae,f). PS is known to be a disease driven by the effects of IL-17A and IL-17F on keratinocytes and other tissue-resident cell types. As expected, strong IL-17 Signaling Pathway enrichment was detected in the analysis using IPA (Fig. 2Aa), and the associated gene sets are shown in Table 1. An even stronger signal of IL-17 was seen in the full UC DEG set (Fig. 2Ab). Among the shared DEGs and top 10 enriched pathways (Fig. 2B,C), IL-17 Signaling was observed in both PS and UC tissues (Fig. 2Ac,d). In UC-specific gene sets (Fig. 2Af), there was also a strong signal for IL-17 signaling, but this signal was attenuated in among PS-specific DEGs.

**Table 1:** DEGs in the shared signature in IL-17 signaling pathway.

IL17 signaling				
Symbol	Entrez gene name	Expr log ratio	Expr p-value	Expr q-value
**UCTR**				
CCL11	C–C motif chemokine ligand 11	1.202	3.31E−08	1.19E−07
CCL2	C–C motif chemokine ligand 2	1.683	9.95E−24	3.17E−22
CCL20	C–C motif chemokine ligand 20	1.613	9.24E−13	5.72E−12
CCL22	C–C motif chemokine ligand 22	1.906	3.89E−15	3.25E−14
CD70	CD70 molecule	2.096	8.64E−27	5.08E−25
CEBPB	CCAAT enhancer binding protein beta	1.176	3.11E−20	5.41E−19
CLCF1	Cardiotrophin like cytokine factor 1	1.183	1.37E−16	4.56E−26
CXCL1	C-X-C motif chemokine ligand 1	3.95	4.65E−45	3.35E−41
CXCL3	C-X-C motif chemokine ligand 3	3.105	7.67E−34	3.76E−36
DEFB1	Defensin beta 1	− 1.696	2.16E−15	1.87E−14
EDA	Ectodysplasin A	− 1.825	2.33E−05	1.54E−23
FOS	Fos proto-oncogene, AP-1 transcription factor subunit	1.748	5.36E−14	3.93E−13
IL11	Interleukin 11	3.059	2.12E−16	2.13E−15
IL33	Interleukin 33	1.741	7.31E−25	3.42E−24
IL1A	Interleukin 1 alpha	3.607	1.84E−25	8.22E−24
IL1B	Interleukin 1 beta	3.264	6.48E−24	2.18E−22
LCN2	Lipocalin 2	3.316	2.94E−26	1.54E−24
LTA	Lymphotoxin alpha	1.566	8.59E−17	9.16E−16
LTB	Lymphotoxin beta	1.45	2.68E−15	2.29E−14
MAPK11	Mitogen-activated protein kinase 11	1.5	1.65E−31	3.13E−29
MMP2	Matrix metallopeptidase 2	1.056	9.15E−09	5.53E−10
MMP3	Matrix metallopeptidase 3	4.764	4.15E−24	1.46E−22
MMP9	Matrix metallopeptidase 9	2.129	5.06E−10	5.32E−19
MRAS	Muscle RAS oncogene homolog	1.167	1.95E−22	9.68E−23
MUC5AC	Mucin 5AC, oligomeric mucusgel-forming	4.087	2.1E−16	1.32E−16
NOS2	Nitric oxide synthase 2	2.714	3.03E−10	3.87E−22
OSM	Oncostatin M	3.476	1.32E−17	1.57E−16
PGF	Placental growth factor	1.38	6.05E−21	1.19E−19
PIK3CD	Phosphatidylinositol-4,5-bisphosphate 3-kinase catalytic subunit delta	1.042	4.57E−14	3.38E−13
PTGS2	Prostaglandin-endoperoxide synthase 2	2.476	6.59E−17	7.14E−16
RGS16	Regulator of G protein signaling 16	1.856	5.13E−26	2.54E−24
TGFB2	Transforming growth factor beta 2	1.591	6.29E−21	1.24E−19
TGFB3	Transforming growth factor beta 3	1.336	4.34E−10	1.54E−16
TNF	Tumor necrosis factor	1.158	2.58E−13	1.72E−12
TNFRSF11B	TNF receptor superfamily member 11b	1.11	1.24E−11	6.62E−11
TNFSF8	TNF superfamily member 8	1.4	1.9E−20	3.44E−19
TNFSF9	TNF superfamily member 9	2.462	3.63E−30	4.63E−28
TNFSF11	TNF superfamily member 11	1.883	8.88E−14	6.31E−13
TNFSF13B	TNF superfamily member 13b	1.098	2.43E−17	2.8E−16
VEGFC	Vascular endothelial growth factor C	1.632	1.01E−35	6.63E−33
**PSTR**				
CDC25A	Cell division cycle 25A	1.057	2.54E−03	2.55E−24
IL7R	Interleukin 7 receptor	1.188	2.78E−04	5.18E−23
LYN	LYN proto-oncogene, Src family tyrosine kinase	1.182	2.40E−44	1.60E−52
SHC1	SHC adaptor protein 1	1.203	3.99E−12	8.00E−38
SLC2A1	Solute carrier family 2 member 1	1.242	2.63E−34	6.02E−33
STAT1	Signal transducer and activator of transcription 1	1.882	3.29E−03	1.93E−56

Expr Log Ratio: log2 FC between lesional and nonlesional samples in PS or UC.

*DEG* differentially expressed gene, *PSTR* psoriasis transcriptome, *UCTR* ulcerative colitis transcriptome.

Indicated by p-values listed in Table 1, the stronger overall enrichment of IL-17 Signaling in UC versus PS is reflected by more pronounced expression changes of individual genes and all of which are much more significant. Most of the gene products identified from the current study as listed in Table 1 have demonstrated biological roles in PS that include induction of innate and adaptive cytokines, as well as induction of antimicrobial proteins. By extension, compared to PS, UC represents an even more inflammatory environment, with strong upregulation of many IL-17-regulated innate cytokines/chemokines and higher expression of some proteases (MMP-3, MMP-9). Other notable pathways that are shared across the two diseases in the current study include Dendritic Cell Maturation, Th1 Pathway, IL-8 Signaling and Leukocyte Extravasation Signaling.

Dysregulated pathways involving DEGs in PS and/or UC were mostly related to Th17/Th1/Th2, which are substantially activated in lesional colon UC tissues (Fig. 2Aa,b). Pathways affected by the shared genes revealed comparable effects in both diseases (Fig. 2Ac,d).

The Estrogen Receptor Signaling Pathway is another pathway that is more strongly induced in UC than in PS, thus providing additional evidence supporting the role of the estrogen receptor in UC-related carcinogenesis^27^. Interestingly, HER-2 Signaling in Breast Cancer and Breast Cancer Regulation in Stathmin1 pathways are both enriched to a higher degree in UC than in PS^28^.

In addition, PPAR Signaling Pathway also exhibits greater repression in UC than in PS. A reported emerging function of PPARs includes a role in maintaining the homeostasis of intestinal tissue^29^. Decara et al. also pointed out that PPARα activation decreases the production of inflammatory cytokines by a variety of cell types^29^.

Another study on the analysis of PPAR signaling activity in PS^30^ tested the hypothesis that low levels of PPAR*g* expression promote the development of psoriatic lesions, triggering the IL-17-related signaling cascade. The results indicated that PPAR*g* decreases the expression of genes that contribute to the development of psoriatic lesions^30^.

Taken together, these findings confirm that PPARs and their ligands have important therapeutic potential^29^.

In parallel, analysis of DEGs from the shared signature that contribute to Role of IL-17A in Psoriasis Pathway revealed greater enrichment in psoriatic lesion skin than in intestinal UC lesional tissues versus the corresponding nonlesionnal samples, as evidenced by the overexpressed chemokines (CCL20, CXCL1, CXCL3, CXCL6) and S100 genes (S100A8, S100A9) (Table 2).

**Table 2:** DEGs in the shared signature present in role of IL-17A in psoriasis pathway.

Role of IL17A in psoriasis		PSTR			UCTR		
Symbol	Entrez gene name	Expr log ratio	Expr p-value	Expr q-value	Expr log ratio	Expr p-value	Expr q-value
CCL20	C–C motif chemokine ligand 20	3.286	1.59E−25	9.83E−39	1.613	9.24E−13	5.72E−12
CXCL1	C-X-C motif chemokine ligand 1	3.776	4.16E−24	5.85E−55	3.95	4.65E−45	3.35E−41
CXCL3	C-X-C motif chemokine ligand 3	1.007	9.32E−20	8.01E−19	3.105	7.67E−34	3.76E−36
CXCL6	C-X-C motif chemokine ligand 6	1.045	8.54E−14	4.66E−13	3.824	1.17E−24	4.58E−23
S100A8	S100 calcium binding protein A8	1.117	1.37E−26	1.92E−25	3.591	1.83E−18	2.46E−17
S100A9	S100 calcium binding protein A9	3.339	1.33E−18	4.26E−54	3.227	4.89E−25	2.03E−23

*PSTR* psoriasis transcriptome defined as expression differences between lesional and non-lesional skin, *UCTR* ulcerative colitis transcriptome defined as expression differences between lesional and non-lesional colon, *Expr Log Ratio* Log Ratio Expr, fold change (FC) in log2 scale, *DEGs* differentially expressed genes.

### Baseline cross-disease correlation between disease activity and diseased tissue expression and pathway activities

Gene expression mapped onto the canonical Hallmark gene sets^31^ and established psoriasis related pathways^20,32^ exhibit significant correlation (rho ≥ 0.3–0.5, p ≤ 0.1) between UC disease scores as measured by Mayo scores at baseline and PS-related pathway/gene set activities. (Fig. 4 and Table 3).

**Table 3:** Established psoriasis pathways significantly correlated (p < 0.05) with Mayo Scores in lesional tissues from UC patients, but not in nonlesional tissues.

Gene set	Inflamed		Non-Inflamed	
	pvalue	Correlation coefficient	pvalue	Correlation coefficient
JK_MACROPHAGE_AHCELLMAPS_UP	4.48E−03	0.5945	6.68E−01	− 0.1086
HALLMARK_MTORC1_SIGNALING	5.58E−03	0.5826	4.20E−01	0.2025
HALLMARK_ANDROGEN_RESPONSE	9.74E−03	0.5503	5.90E−01	0.136
HALLMARK_UNFOLDED_PROTEIN_RESPONSE	2.25E−02	0.4949	5.11E−01	0.1656
JK_KC_IL17_AND_TNF_DOWN	2.87E−02	0.4772	5.99E−01	0.1329
HALLMARK_UV_RESPONSE_UP	3.77E−02	0.4561	6.20E−01	− 0.1255
JK_MPH_IFNG_UP	4.30E−02	0.4455	9.24E−01	− 0.0243
JK_MONOCYTES_IL17_UP	4.51E−02	0.4416	3.17E−01	− 0.2499
JK_PSORIASIS_NGS_UP	4.54E−02	0.4409	7.33E−01	0.0865
JK_MATURE_DC_AHCELLMAPS_UP	4.73E−02	0.4376	5.03E−01	− 0.1687
JK_MAD3_PSORIASIS_UP	4.80E−02	0.4363	9.34E−01	0.0211
JK_FIBROBLASTS_IL17_UP	4.91E−02	0.4343	6.41E−01	0.1181

**Figure 3:**
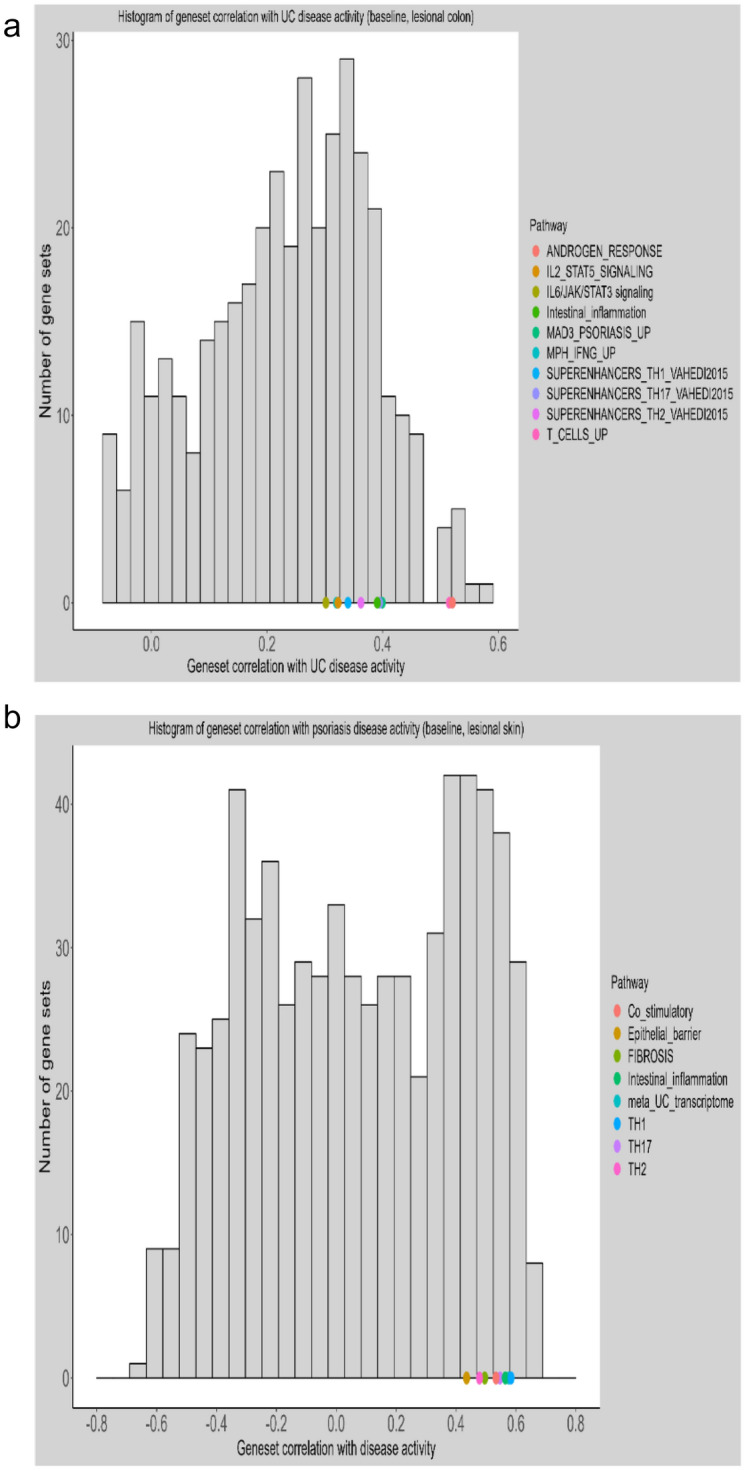
Baseline cross-disease correlation between disease activity and pathway activities. (**a**) Psoriasis related pathways are positively correlated with Mayo Scores of UC patients (lesional colon at baseline); Y axis indicates # of pathways/gene sets; X axis is correlation coefficient between pathway/gene set activity and UC disease activity as represented as Mayo Score. (**b**) Pathways dysregulated in UC related are positively correlated with disease scores (PASI) of psoriasis patients (lesional skin at baseline); Y axis indicates # of pathways/gene sets; X axis is correlation coefficient between pathway/gene set activity and psoriasis disease activity as represented as PASI.

In parallel, pathways/gene sets with established roles in UC pathophysiology were positively correlated (rho ≥ 0.4–0.6, p ≤ 0.1) with Psoriasis Area and Severity Index (PASI) in lesional skin tissues from PS patients. These pathways include epithelia barrier, general inflammation, Th1, Th2 and Th17 (Fig. 3).

Of note, genes in the shared signature significantly correlated (p ≤ 0.05) with UC disease activities (Mayo scores) are underscored by fatty acid metabolism as suggested by FADS1, FADS2 (Table 4). Studies provide evidence that FFA amplifies pro-inflammatory response and psoriatic inflammation^33^.

**Table 4:** Genes in the shared signature significantly correlated (p < 0.05) with UC disease activities (Mayo scores).

Symbol	UC inflamed vs. non-inflamed			Inflamed		Non-inflamed	
	log2FC	pvalue	fdr	pvalue	Correlation coefficient	pvalue	Correlation coefficient
FADS1	1.72	1.97E−16	8.78E−28	0.00E+00	0.61	3.70E−01	0.23
FADS2	2.13	2.92E−10	3.14E−22	1.00E−02	0.56	1.60E−01	0.35
CHI3L2	2.63	1.91E−22	4.86E−21	1.00E−02	0.54	3.30E−01	− 0.24
FIBIN	1.82	3.04E−18	3.96E−17	1.00E−02	0.54	3.10E−01	0.25
CH25H	1.32	5.22E−18	6.52E−17	1.00E−02	0.53	7.20E−01	0.09
HSD11B1	1.95	3.41E−13	1.62E−15	3.00E−02	0.48	5.00E−02	0.47
CXCL6	3.82	1.17E−24	4.58E−23	4.00E−02	0.45	3.00E−02	0.5
SLC7A5	1.61	4.44E−22	1.06E−20	4.00E−02	0.45	2.70E−01	0.27
TESC	1.83	1.35E−32	3.89E−30	4.00E−02	0.45	4.80E−01	− 0.18

To establish reasonable baselines for the comparisons between UC RNA sequencing (RNA-seq) data and PS microarray data in the current study, the sensitivity analysis of 3 published UC microarray datasets (GSE38713, GSE23597, GSE16879)^34^ revealed 83% unique genes in both UC and PS and 17% shared genes, consistent with findings from the current study. In addition, the 50 upregulated genes in PS include most of the top 50 upregulated shared genes identified in this study (Table 5).

**Table 5:** Top 50 most up-regulated among shared genes between Psoriasis (NTC03210961) and UC (GSE38713-GSE23597-GSE16879)^34^.

Symbol	Lesional vs. non-lesional															
	PS microarray from C250_1001				UC microarray from GSE38713-GSE23597-GSE16879				Current PS microarray metadata				Current UC RNAseq metadata			
	log2FC	FC	p-value	fdr	log2FC	FC	p-value	fdr	log2FC	FCH	p-value	fdr	log2FC	FC	p-value	fdr
CXCL13	3.83	14.23	5.18E−31	4.07E−28	3.52	11.5	6.18E−05	1.32E−03	3.69	12.94	1.05E−86	4.60E−84	3.33	10.05	2.50E−13	1.67E−12
TCN1	3.81	14.03	1.14E−25	9.59E−24	5.95	62.03	6.68E−24	5.31E−20	4.37	20.67	1.34E−67	2.01E−65	5.2	36.79	1.11E−37	1.23E−34
CXCL1	3.51	11.39	2.81E−23	1.29E−21	4.94	30.66	1.04E−24	1.39E−20	3.78	13.7	4.16E−24	5.85E−55	3.95	15.46	4.65E−45	3.35E−41
TNIP3	3.4	10.56	7.64E−28	1.42E−25	3.86	14.47	2.39E−07	1.28E−05	3.37	10.35	5.44E−55	4.32E−53	3.92	15.14	5.41E−26	2.67E−24
KYNU	2.93	7.63	3.50E−35	3.22E−31	2.64	6.25	9.02E−11	1.65E−08	3.51	11.41	4.70E−143	3.29E−139	2.42	5.35	8.92E−35	4.14E−32
VNN1	2.81	7.01	1.23E−31	1.37E−28	2.86	7.26	7.23E−08	4.58E−06	3.22	9.33	6.47E−27	1.82E−98	3.72	13.15	1.08E−17	7.15E−25
GZMB	2.76	6.75	1.17E−27	2.03E−25	1.94	3.83	4.41E−06	1.45E−04	3.13	8.75	4.60E−53	4.73E−71	1.91	3.75	2.07E−14	2.08E−19
CCL20	2.72	6.57	2.50E−19	4.98E−18	2.1	4.29	1.71E−06	6.66E−05	3.29	9.76	1.59E−25	9.83E−39	1.61	3.06	9.24E−13	5.72E−12
MMP1	2.54	5.82	6.15E−15	5.77E−14	4.84	28.74	4.31E−11	8.64E−09	2.89	7.39	7.10E−24	8.21E−23	2.88	7.34	3.95E−17	4.43E−16
MMP12	2.54	5.81	1.21E−16	1.49E−15	4.17	18.02	1.67E−20	6.90E−17	2.98	7.9	1.84E−23	1.08E−33	2.26	4.79	2.07E−24	7.82E−23
OAS2	2.46	5.5	1.32E−25	1.10E−23	1.17	2.24	4.67E−05	1.04E−03	2.85	7.23	1.85E−51	3.47E−87	1.28	2.44	6.59E−22	2.95E−23
CXCL10	2.39	5.23	3.15E−20	7.39E−19	2.58	5.98	1.08E−06	4.55E−05	2.59	6.04	1.97E−22	4.65E−34	1.99	3.96	1.05E−12	6.45E−12
EPSTI1	2.35	5.12	5.09E−27	6.86E−25	0.74	1.67	1.45E−03	1.77E−02	2.78	6.86	2.73E−26	6.56E−101	0.57	1.49	5.03E−06	6.39E−08
SAMD9	2.35	5.08	5.06E−32	7.28E−29	0.62	1.54	3.44E−03	3.52E−02	2.71	6.55	1.51E−31	2.28E−100	0.46	1.37	5.37E−04	6.29E−04
CXCL2	2.33	5.04	3.04E−24	1.74E−22	3.73	13.23	5.93E−14	3.45E−11	1.77	3.42	8.66E−02	8.42E−05	3	8.02	1.47E−25	1.19E−31
ISG15	2.29	4.91	3.12E−24	1.79E−22	0.97	1.96	1.38E−04	2.56E−03	2.6	6.07	9.79E−65	1.34E−62	0.99	1.98	9.59E−12	5.20E−11
HPSE	2.18	4.52	4.06E−26	3.95E−24	0.82	1.76	1.76E−04	3.14E−03	2.69	6.45	4.66E−116	1.09E−112	0.55	1.46	1.01E−11	5.46E−11
CCL3	2.1	4.3	7.67E−20	1.67E−18	1.83	3.56	4.38E−03	4.27E−02	1.62	3.07	1.26E−05	2.17E−25	1.9	3.74	2.39E−14	1.83E−13
ZC3H12A	2.1	4.27	5.13E−29	1.46E−26	2.47	5.54	6.25E−13	2.32E−10	2.02	4.05	7.83E−30	7.15E−67	1.37	2.59	1.23E−19	2.33E−23
CXCL9	2.09	4.25	3.64E−19	7.03E−18	3.42	10.68	2.86E−10	4.34E−08	2.3	4.94	3.58E−27	3.38E−28	2.32	4.99	3.36E−17	3.81E−16
LCN2	2.07	4.19	3.76E−21	1.07E−19	4.56	23.52	5.27E−21	2.35E−17	2.71	6.56	1.96E−53	1.43E−51	3.32	9.96	2.94E−26	1.54E−24
S100A9	2.06	4.17	8.08E−17	1.02E−15	4.01	16.15	1.70E−10	2.85E−08	3.34	10.12	1.33E−18	4.26E−54	3.23	9.36	4.89E−25	2.03E−23
IFI6	2.03	4.08	1.33E−20	3.34E−19	1.57	2.97	1.32E−07	7.67E−06	2.43	5.39	5.53E−79	1.45E−76	1.43	2.7	4.89E−22	1.16E−20
IDO1	1.99	3.97	9.13E−31	6.32E−28	3.92	15.18	1.25E−11	2.96E−09	2.44	5.44	1.36E−50	8.41E−83	3.02	8.1	1.65E−22	4.27E−21
BCL2A1	1.94	3.84	6.95E−23	2.92E−21	2.67	6.37	1.12E−05	3.16E−04	2.21	4.64	4.19E−23	7.54E−47	2.02	4.06	2.14E−18	2.84E−17
IL1B	1.92	3.78	3.07E−17	4.19E−16	3.58	11.94	3.96E−10	5.63E−08	1.91	3.76	3.82E−12	3.90E−20	3.26	9.6	6.48E−24	2.18E−22
MX1	1.88	3.69	4.85E−25	3.44E−23	0.74	1.67	3.67E−03	3.71E−02	2.14	4.42	2.25E−32	2.44E−96	0.39	1.31	1.01E−03	2.01E−03
RND1	1.86	3.64	1.38E−23	6.82E−22	1.17	2.25	1.18E−03	1.49E−02	1.74	3.34	2.14E−22	1.35E−35	1.63	3.08	3.44E−15	1.08E−15
CD80	1.83	3.55	2.70E−28	5.95E−26	1.32	2.49	1.13E−05	3.18E−04	1.73	3.31	1.30E−41	4.85E−40	1.51	2.85	4.36E−18	5.52E−17
RTP4	1.81	3.52	9.00E−27	1.09E−24	0.84	1.79	4.24E−05	9.63E−04	2.19	4.56	3.27E−26	1.06E−101	0.6	1.52	1.64E−13	1.12E−12
CCL4	1.81	3.52	6.90E−25	4.67E−23	2.39	5.23	1.34E−06	5.50E−05	1.81	3.5	4.57E−15	8.24E−54	1.78	3.43	6.37E−17	6.91E−16
FCGR1B	1.78	3.42	5.54E−21	1.51E−19	2.33	5.01	2.65E−06	9.59E−05	1.45	2.72	1.07E−07	1.40E−21	1.96	3.89	2.16E−15	1.87E−14
WNT5A	1.76	3.38	5.58E−30	2.63E−27	2.08	4.24	3.58E−06	1.23E−04	2.08	4.21	4.62E−83	1.44E−91	1.19	2.29	1.06E−11	5.72E−11
PI3	1.74	3.34	9.80E−15	8.93E−14	4.19	18.24	1.44E−19	3.87E−16	2.07	4.2	5.21E−34	4.92E−35	4.26	19.16	7.93E−11	1.35E−33
LIPG	1.74	3.34	1.99E−14	1.73E−13	1.54	2.9	1.05E−09	1.32E−07	2.11	4.31	1.63E−29	2.76E−28	0.89	1.85	6.56E−16	6.14E−15
CXCL11	1.73	3.33	6.53E−15	6.10E−14	2.42	5.36	8.07E−05	1.64E−03	1.4	2.63	1.74E−19	1.29E−20	3.03	8.16	4.77E−18	5.99E−17
CXCR2	1.72	3.3	7.14E−28	1.34E−25	2.24	4.73	2.46E−04	4.14E−03	1.84	3.58	5.45E−29	1.32E−53	3.32	9.96	4.38E−20	7.45E−19
PLAT	1.71	3.28	1.66E−18	2.85E−17	1.28	2.43	8.40E−06	2.48E−04	1.83	3.56	7.58E−37	2.07E−35	0.09	1.06	4.99E−01	5.64E−01
APOL1	1.71	3.27	1.88E−25	1.51E−23	1.68	3.19	5.85E−07	2.71E−05	1.87	3.65	7.79E−34	4.68E−78	1.84	3.58	4.60E−14	9.96E−28
CASP5	1.71	3.26	1.09E−19	2.30E−18	1.61	3.05	7.77E−06	2.32E−04	2.17	4.51	1.09E−04	1.02E−45	1.01	2.01	3.22E−08	4.02E−09
FCGR1A	1.66	3.17	8.19E−21	2.16E−19	1.76	3.38	3.55E−05	8.35E−04	1.65	3.13	6.20E−05	3.37E−25	1.79	3.46	1.05E−16	1.11E−15
LRP8	1.65	3.14	1.00E−30	6.63E−28	0.74	1.67	2.09E−03	2.37E−02	1.58	2.99	3.06E−52	2.10E−50	1.76	3.39	6.45E−22	5.44E−29
XDH	1.64	3.13	5.29E−22	1.80E−20	1.47	2.77	1.45E−05	3.92E−04	1.7	3.25	5.59E−09	1.34E−31	0.83	1.78	8.57E−07	1.71E−06
OAS3	1.64	3.12	1.07E−25	9.09E−24	0.78	1.72	4.85E−05	1.07E−03	2.04	4.1	1.53E−95	1.07E−92	0.52	1.43	5.71E−04	3.16E−07
TDO2	1.59	3.02	4.15E−18	6.62E−17	2.3	4.92	5.69E−05	1.23E−03	1.65	3.13	2.69E−24	3.21E−23	2.07	4.2	4.22E−21	8.57E−20
CHI3L2	1.57	2.98	3.58E−16	4.09E−15	2.47	5.54	4.13E−07	2.03E−05	1.77	3.41	1.23E−27	1.84E−26	2.63	6.17	1.91E−22	4.86E−21
IRF7	1.55	2.92	3.57E−27	5.11E−25	0.68	1.6	6.06E−04	8.66E−03	1.73	3.32	9.69E−31	3.59E−69	0.42	1.34	1.48E−05	3.80E−05
FPR1	1.54	2.9	1.64E−17	2.35E−16	2.91	7.54	7.05E−07	3.18E−05	1.03	2.04	1.10E−01	8.47E−02	3.16	8.97	6.71E−21	1.31E−19
MX2	1.53	2.89	6.14E−21	1.66E−19	2.12	4.33	4.72E−08	3.23E−06	1.67	3.19	7.79E−16	1.66E−59	1.05	2.06	3.82E−14	2.84E−13
SLAMF7	1.53	2.88	4.77E−24	2.63E−22	1.32	2.5	9.25E−04	1.22E−02	1.66	3.16	1.63E−71	3.03E−69	0.99	1.99	2.39E−15	2.05E−14

### The PS-UC shared tissue signature demonstrates a good correlation with molecular improvement and clinical efficacy

To determine the clinical utility of the shared signature, we calculated a gene set improvement score using this shared signature in the context of Pfizer clinical studies of therapies for PS [NCT02370150 (Phase 1, TYK2/JAK1 inhibitor Brepocitinib, PF-6700841)]^35^ and UC [NCT01620255 (Phase 2b, anti-MAdCAM antibody, PF-00547659)]^36^.

**Figure 4:**
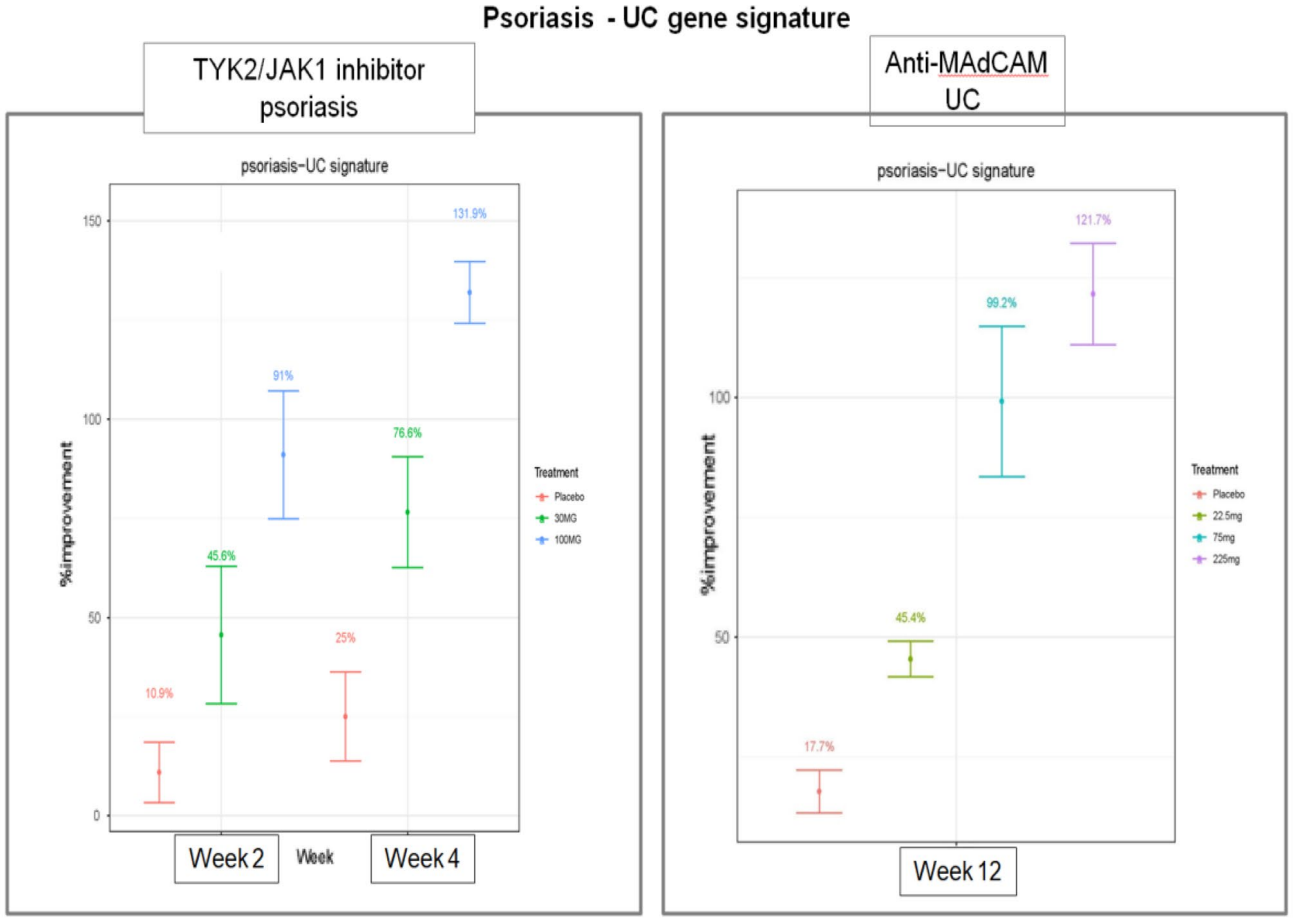
The shared tissue signature demonstrates % molecular improvement dose dependently in PS and UC patients after respective treatment.

Among patients with moderate to severe disease, the shared signature genes in lesional tissues returned toward nonlesional levels in a dose-dependent manner with PF-00547659 (anti-MAdCAM) in UC patients^36^ and PF-6700841 (TYK2/JAK1) in PS patients^35^, as shown in Fig. 4.

### Validating the PS-UC disease signature with a permutation test

The statistical significance of the gene signature response was assessed by permutation of genes. We randomly extracted 20 gene sets of comparable size as the meta-analysis-defined PS-UC gene signature and calculated an improvement score for each gene set using the Brepocitinib Phase 2 PS study (NCT02969018)^35^ and the anti-MAdCAM Phase 2 UC study (NCT01620255)^36^. The improvement scores based on the 20 randomly generated gene sets did not show any trends in terms of treatment effect, suggesting that the evident dose-dependent effect on the shared gene signature score defined by meta-analysis is not random.

## Discussion

The association between PS and UC was first investigated in epidemiological studies^37^. A meta-analysis of 5 case–control or cross-sectional studies (# of participants = 1,826,777) and 4 cohort studies (# of participants = 5,967,410)^38^ found that patients with PS were 1.75 times more likely to develop UC than controls. Likewise, the cohort studies showed increased odds ratios (PS versus controls) of 2.53 for Crohn’s disease and 1.71 for UC^38^. Another study found that IBD patients were also more likely to develop PS than non-IBD subjects^36^.

In this study, we identified a shared tissue gene expression signature from 3 Pfizer clinical transcriptomic studies on PS (Microarray) and 3 UC studies based on estimates of differences in baseline lesional and nonlesional tissues.

Of note, among genes shared in UC and PS, 155/190 demonstrated consistent direction of changes. These included the interferon-induced genes IFI6, CXCL9, CXCL10 and CXCL11; IL-23A, CCL20, PI3, CXCL1 and LCN2 in the IL-23 pathway, and the costimulatory molecules ICOS and CTLA4, which are suggestive of ongoing T-cell activation in both diseases (Fig. 1D,E).

The pathway analysis also showed that cytokine response pathways largely behave similarly across both diseases, as suggested by the activation of shared key proinflammatory cytokines, including IL-23, IL-6, IL-8, IL-7, and IL-12, Th17 (other than IL-17A) and anti-inflammatory cytokines, such as IL-10, which are known for their involvement in the pathophysiology of both diseases. Interestingly, Tumor Microenvironment and Colorectal Cancer Metastasis Signaling pathways exhibited more significant induction in UC than in PS, consistent with the reported increased risk of colorectal cancer in UC patients^27^.

Our study also elaborates on the general implications of the Th17 pathway in both psoriatic skin and colon lesional tissues. In support of the Role of IL-17A in the Psoriasis Pathway, CXCL1, CXCL3, CXCL6, S100A8, S100A9, and CCL20 in the shared signature were all upregulated in both psoriatic skin and colon lesional tissues.

Elevated IL-17A levels in UC have been reported in several studies^39,40^. Nonetheless, Th17 signature cytokine, IL-17A may target gut epithelial cells and promote the activation of regulatory pathways that give protection in the gastrointestinal tract^41,42^. To this end, anti-IL-17A clinical trials in IBD showed no therapeutic benefits, with increased adverse events in the treatment group^41^. This cause concerns about the role of IL-17A in IBD pathogenesis and suggested that the elevated IL-17A might be beneficial for IBD patients^41^.

In our study, IL-17A/F/C/D exhibited significantly increased expression in psoriatic skin lesions compared to nonlesional skin, but not in colon lesion tissues versus nonlesional tissues from UC patients. Consequently, IL-17A/F/C/D and/or IL-17RA and its isoforms are not part of the shared PS-UC signature. These findings are supported by the differential efficacy of IL-17 inhibitors for PS and UC. The three currently available monoclonal antibody therapies targeting IL-17A (Secukinumab and Ixekizumab)^43–46^, or IL-17 receptor A (Brodalumab)^47^ have been proven effective in the treatment of PS. These efficacy studies prompted the evaluation of anti-IL17 therapies in IBD, where some paradoxical events, such as disease exacerbation, were observed^48^. The differential efficacy of IL-17 inhibitors seems to be based on the cytokine variant that is selectively inhibited by a specific treatment^22^. IL-17A and IL-17F share high sequence homology but perform different functions^49^. IL-17A is involved in inflammation and autoimmunity, while IL-17F is predominantly involved in mucosal defense mechanisms^50^. IL-17A has demonstrated differential effects in PS and IBD: in psoriasis, it is overexpressed in baseline psoriatic skin lesions as a primary driver of the disease pathology, while in IBD, it promotes the activation of regulatory pathways and confers protection in the gastrointestinal tract^51^. Thus, inhibition of IL-17A may interfere with the protective function of IL-17A in the intestine, as evidenced by the adverse outcomes in the case studies with IL-17A inhibitors in IBD^51,52^. The above findings confirm the hypothesis of different functional effects of IL-17A in PS and IBD, including UC.

The gene set improvement score (GSI) of the PS-UC shared gene signature illustrates dose-dependent molecular improvement in gene expression in the PF-00547659 (anti-MAdCAM antibody) phase 2 study in patients with moderate to severe UC^37^ and the PF-6700841 (a TYK2/JAK1 inhibitor Brepocitinib) clinical study in patients with moderate to severe PS^35^. The marked molecular improvement demonstrated by the shared signature is consistent with the clinical efficacy of these drugs in both diseases, as measured by % improvement score (a post-therapy metric of molecular response).

## Conclusion

This is the first comparison study to use meta-analysis of tissue transcriptome profiling to define a robust shared gene signature and to investigate dysregulated disease-related pathways common to both PS and UC.

Our study identified a signature consisting of overlapping genes as key players driving mucosal inflammation in UC and skin inflammation in PS, indicating these genes reflecting inflammatory status in both diseases. The absence of IL-17A from the shared signature underscores its different functional effects in PS versus UC, as reflected by the exclusive therapeutic benefit of IL-17A-targeting agents in PS.

Furthermore, the shared gene signature demonstrates a good correlation with molecular improvement and clinical efficacy in both PS and UC as measured by the percent improvement following treatments with different mechanisms of action. The fact that this shared signature has been shown to capture therapeutic effects of different drug mechanisms of action suggests the potential for broader clinical utilities and applications.

## Materials and methods

### Selected datasets

Transcriptomic data from a total of 6 Pfizer clinical studies between 2012 and 2018, 3 on PS and 3 on UC (Table 6), were selected for a meta-analysis to define a shared tissue signature. The disease characteristics reported were the Psoriasis Area and Severity Index (PASI) score for the PS studies and the total Mayo score (considering stool frequency, rectal bleeding, endoscopic evaluation, and physician's assessment) for the UC studies.

**Table 6:** Studies used in the meta-analysis.

Dataset(s)	Sequencing platform	Sample size P: # of patients N: # of samples	Disease	Time point	Tissue type
NCT02310750 (PF-06700841)	Affymetrix human genome U133 plus 2.0 array	P = 30, N = 60	Psoriasis	Baseline	Skin lesional, non-lesional (paired)
NCT03210961(PF-06826647)	Affymetrix human genome U133 plus 2.0 array	P = 40, N = 80	Psoriasis	Baseline	Skin lesional, non-lesional (paired)
NCT01710046 (CP-6890, 550)	Affymetrix human genome U133 plus 2.0 array	P = 12, N = 24	Psoriasis	Baseline	Skin lesional, non-lesional (paired)
NCT02840721 (PF-06480605)	RNA-seq	P = 39, N = 78	Ulcerative colitis	Baseline	Colon lesional, non-lesional (paired)
NCT01620255 (PF-00547659)	RNA-seq	P = 70, N = 140	Ulcerative colitis	Baseline	Colon lesional, non-lesional (paired)
Unpublished Pfizer internal collection	RNA-seq	P = 18, N = 36	Ulcerative colitis	Baseline	Colon lesional, non-lesional (paired)

UC studies with TL1A Inhibitor PF-06480605 (NCT02840721) and MAdCAM Inhibitor PF-00547659 (NCT01620255) included both male and female patients with mean age around 40 years old; slightly more males than females, more white patients than other races, and moderate to severe active UC measured by Total Mayo Score. The 3rd UC study included in the manuscript was an observational study (unpublished) consisting of patients with initial IBD diagnosis and/or with a flare on standard of care medications. This UC cohort shares similar mean age, sex and race ratios as PF-06480605 and PF-00547659 studies (NCT01620255 and NCT02840721). The mean baseline Total Mayo Scores for the 3 studies were 9.4 (NCT02840721), 8.46 (NCT01620255) and 5 (unpublished observational study). Patients’ mean BMI are 23.6 (kg/m^2^) for NCT02370150 and 24.98 (kg/m^2^) for NCT03210961, while relevant data is not available for the unpublished observation study.

In the two UC clinical trial studies, concomitant medications of low dose steroids, 5-ASA and immunomodulators (AZA/6-MP) were allowed. Biologics such as anti-TNF and anti-integrin were not. For the unpublished observational study, all SOC (Standard of Care) medications were acceptable.

The 3 PS studies, PF-06700841 (TYK2/JAK1 Inhibitor, NCT02370150), PF-06826647 (TYK2 Inhibitor, NCT03210961), PF-00547659 (Tofacitinib, NCT01710046) included both male and female patients with mean age around mid-40’s, predominately white, with moderate to severe disease activity at the time of enrollment (mean PASI of 20.8 (NCT02370150), 27.32 (NCT01620255) and 19.2 (NCT03210961). Patients’ mean BMIs ranged from 31.9 (kg/m^2^) (NCT02370150), 22.32 (kg/m^2^) (NCT03210961) and 28.7 (kg/m^2^) (NCT01710046).

Whole transcriptome data from tissue samples collected in a controlled clinical trial is not widely available. These studies were chosen because they were the full data set available to the authors (at Pfizer) at the time. These studies are considerably larger than any equivalent datasets having full transcriptome in the public domain. The UC studies and data was designed and generated in Pfizer, while PS studies were designed by Pfizer and data was analyzed by Rockefeller University under the supervision of Professor James G. Krueger (co-author on this manuscript). The use of Microarray vs. RNA-seq technology represent the evolving choice of best technology available at the time, however, the technology used is consistent within each disease.

Reviewed by Pfizer Medical Sub-Committee, all methods were carried out in accordance with relevant guidelines and regulations; all experimental protocols were approved by Pfizer Medical Sub-Committee and informed consent was obtained from all subjects and/or their legal guardian(s).

### Preprocessing and gene expression analysis

Within NCT02840721, NCT01620255, unpublished Pfizer internal collection clinical studies, transcriptomic expression profiles from colon biopsy samples collected from patients with moderate to severe UC were examined using RNA-seq technology. The data also included exon and transcript level measures. The GENCODE V25 database was used for annotation (GENCODE Release, Oct. 2016). Biopsy collection for each subject included both an inflamed and a matched non-inflamed biopsy collected prior to treatment.

Additionally, in NCT02370150, NCT03210961, NCT01710046, the gene expression data of skin biopsies from patients with moderate to severe PS were generated using Affymetrix HGU133plus2.0 microarray. The hybridization strategy was performed in concordance with experimental design principles; for example, all samples from the same patient were analyzed on the same date, and samples from every dose group were always included. The data were generated by J. Krueger’s lab at Rockefeller University, utilizing the same preprocessing and analytic procedures across all studies.

For each individual study, transcriptional differences between lesion and nonlesional psoriatic skin or UC colon biopsies were calculated under the general framework for linear models using the LIMMA package (microarray data)^53^ and LIMMA + voom (RNA-seq) packages^54^.

### Meta-analysis

An inverse-variance weighted meta-analysis was performed to determine the effect size of each gene within the combined data from multiple PS and UC studies. For the combined set of studies in each disease, expression differences between lesion and non-lesion biopsies and associated p values were estimated^54^. Bonferroni correction was used to adjust for multiple testing^54–56^. The statistical significance was assessed to estimate the combined FC. DEGs for PS and UC, which represent core disease pathogenesis-related genes, were defined, and termed as PS transcriptome and/or UC transcriptome.

QQ plots generated from p values associated with the heterogeneity test for between-study variations within the same disease suggest minimal *heterogeneity across studies,* with significant heterogeneity in 5% of genes among 3 UC studies and ~ 25% of genes among PS studies.

### Sensitivity analysis

To establish reasonable baselines for the comparisons between UC RNA sequencing (RNA-seq) data and PS microarray data in the current study, a sensitivity analysis of 3 published UC microarray datasets (GSE38713, GSE23597, GSE16879)^34^ was performed, which revealed 83% unique genes in both UC and PS and 17% shared genes, consistent with the current study.

### Correlation analysis

Cross-disease correlation between PASI scores in PS, Mayo scores in UC and disease-specific pathway/gene sets activities was evaluated respectively using Pearson and Spearman correlation coefficients. Results are presented in histograms with estimated correlation coefficients shown in x-axis. Pathway/gene sets significantly correlated with disease scores are colored.

### Gene set improvement score

To demonstrate the shared tissue signature’s clinical utility and correlation with molecular improvement and clinical efficacy, a gene set improvement score was calculated and defined as follows:

for genes up-regulated in lesional tissue—nonlesional tissue, per gene set:

$${\text{GSI }} = { 1}00 \, \times \, \left( {{\text{PT}}\left( {{\text{lesion}}} \right) \, {-}{\text{ BL}}\left( {{\text{lesion}}} \right)} \right)/{\text{Avg}}\left( {\text{non-lesion}} \right) - {\text{BL}}\left( {{\text{lesion}}} \right))$$

where, BL = baseline; PT = post treatment; Avg = average across subjects.

Note: Each per-sample term above is the average of log(expression) across genes in the gene set. While no imbalance of missingness was observed between paired samples within a subject, averages of differences (instead of differences of averages) should be used when this occurs. Genes downregulated in lesion vs. non-lesion can be added into the sum by reversing the sign (i.e., multiplying by − 1) of their log(expression).

### Statistical software and packages

The statistical analysis was carried out in the R language version 3.6.1 (http://www.r-project.org), and packages were from the Bioconductor project (http://www.bioconductor.org).

### Pathway enrichment analysis

Pathway enrichment analysis was performed using IPA (QIAGEN Inc., https://www.qiagenbioinformatics.com/products/ingenuity-pathway-analysis) to further investigate the potential mechanisms underlying each disease in the context of known biological responses and regulatory pathways. Analyses were performed for DEGs passing significance cutoffs, i.e., FDR < 0.05 and |FCH| > 2. The *P* values for enrichment were calculated using a right-tailed Fisher’s exact test to determine statistically significant upregulation/downregulation of genes in the enriched pathways. Z scores reported by IPA for each pathway were used to predict which pathways could be responsible for the activation/inhibition of genes implicated in the diseases.

## Supplementary Information

Supplementary Information.

## Data Availability

All data generated or analyzed during this study are included in this published article (and its Supplementary Information Files).
