# Supplementary material for: A shared tissue transcriptome signature and pathways in psoriasis and ulcerative colitis
Source: Sci Rep. 2022 Nov 17;12:19740. doi: 10.1038/s41598-022-22465-w (PMC9671879; doi:10.1038/s41598-022-22465-w)
Supplement: Supplementary file 1 — Supplementary Information. [file 41598_2022_22465_MOESM1_ESM.docx]

**SUPPLEMENTARY MATERIAL**

**A Shared Tissue Transcriptome Signature and Pathways in Psoriasis and Ulcerative Colitis**

Li Xi^1^, Sandra Garcet^2^, Zhan Ye^1^, Kenneth Hung^1^, Mina Hassan-Zahraee^1^, Elizabeth Kieras^1^, James G Krueger^2^, Craig Hyde^1^, Elena Peeva^1^*

^1^Pfizer, Cambridge, Massachusetts, USA.

^2^Rockefeller University, New York, New York, USA.

^*^Pfizer, Cambridge, Massachusetts, USA.

Electronic address: Elena.Peeva@pfizer.com.


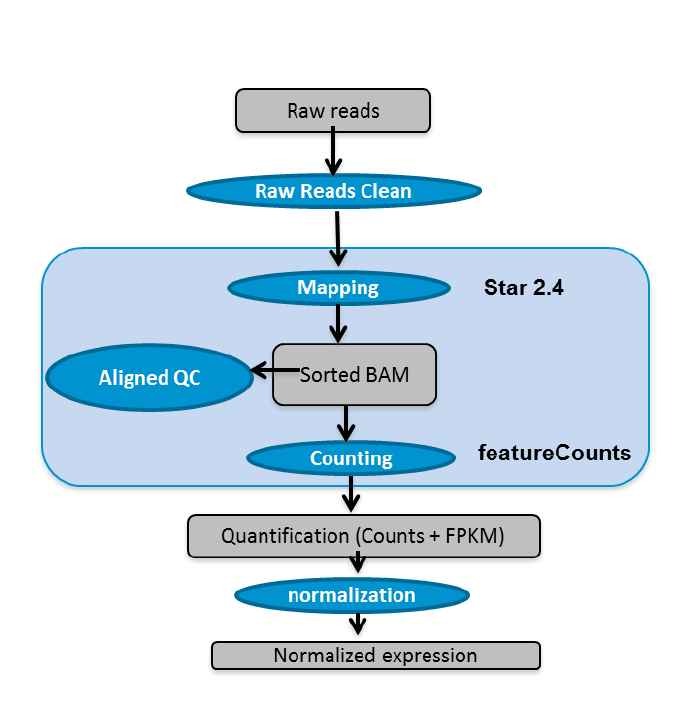


Figure S1. RNAseq preprocessing workflow

**Meta-analysis**

*Reliable signature discovery methods could yield valuable insight into immune cell biology and mechanisms of disease*

The use of Venn-diagrams to identify the intersection of multiple transcriptomic studies has been among the most frequently used approaches. Nevertheless, the caveat is that DEG lists defined by each study differ in aspects including variations in the disease itself (Krueger JG, et al, 2010). As the number of studies increases, it usually results in very few DEGs among studies under comparison, making it difficult if not impossible to identify a set of core feature representing disease. On the other hand, meta-analysis gives each gene in each disease the most robust, maximally powered assessment possible. Intersecting less powered studies will miss otherwise reproducible genes which happen to have low signal in any one study.

*Heterogeneity across studies is minimal*


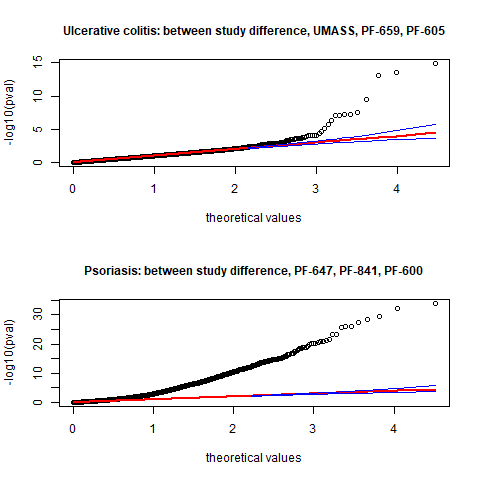


Figure S2 The QQ plots generated from pvalues from heterogeneity test for between study variations, show that the three UC studies only ~5% of genes with het(p) < 0.05, suggesting 95% of the genes profiled, the between study difference is minimal.

For psoriasis studies, the QQ plot from between study heterogeneity test exhibit ~25% of genes with het(p) < 0.05, suggesting 75% of the genes profiled, the between study difference is insignificant.


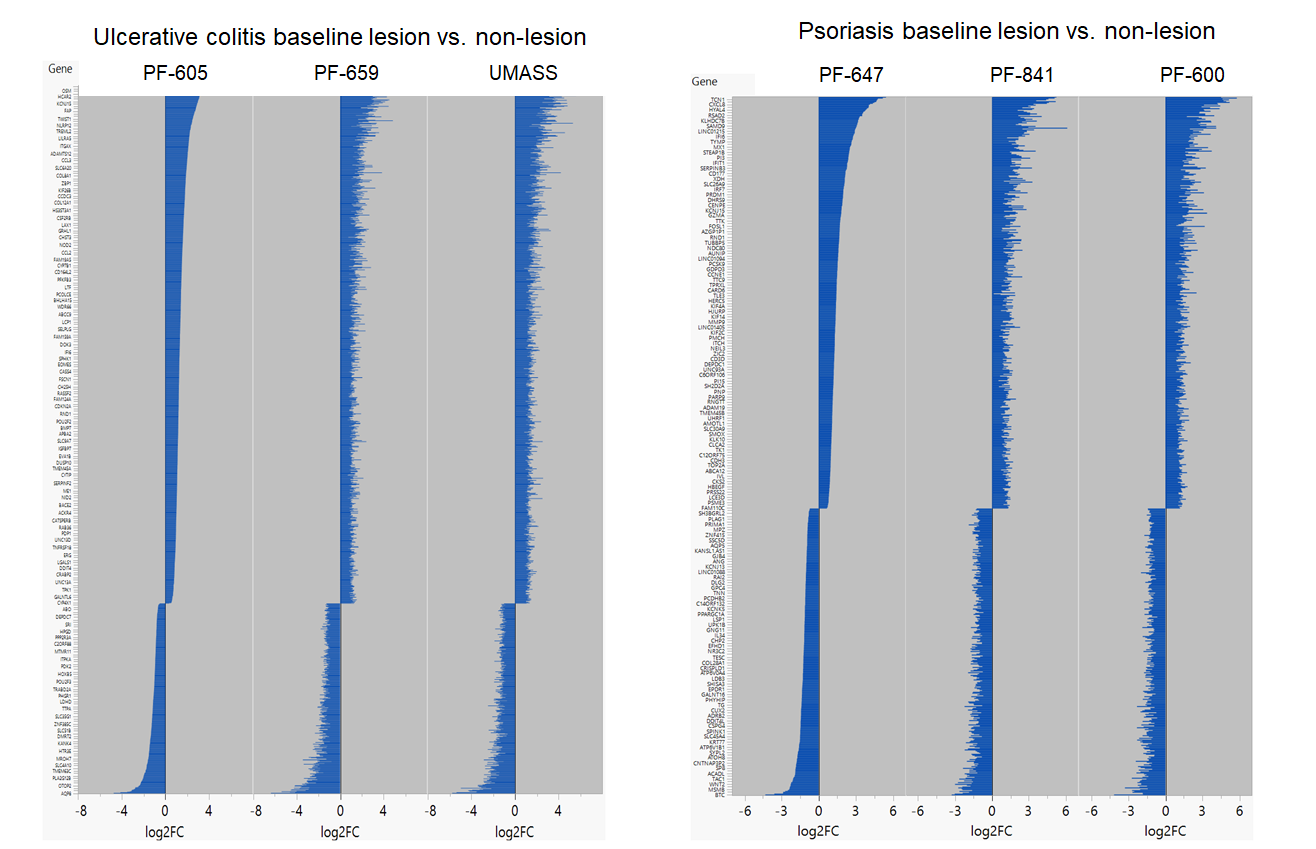


Figure S3 Complete agreement was observed in direction of changes of the identified DEGs among studies in both UC and psoriasis meta-analysis, and magnitude of changes is comparable across studies.


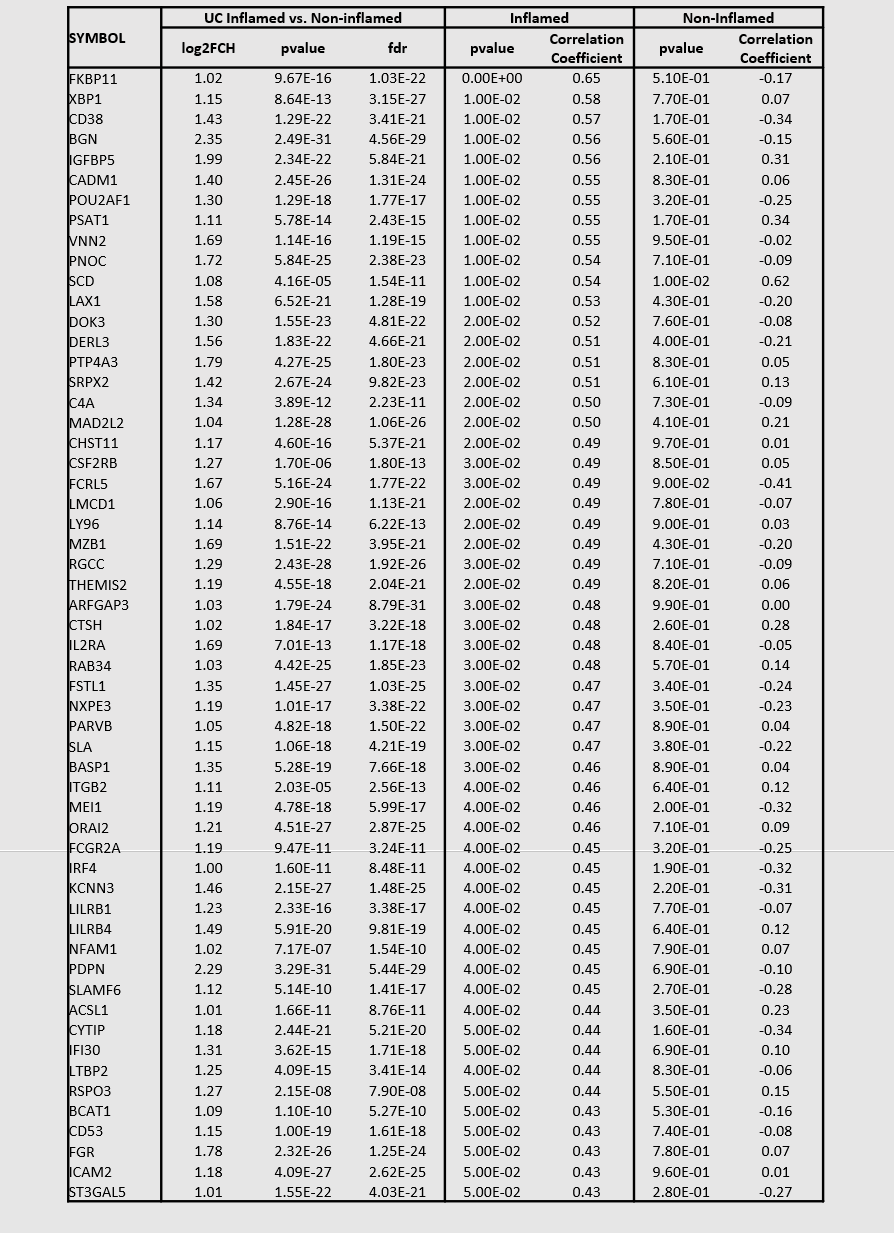


Table S1 Genes significantly correlated with lesional intestinal biopsies (p < 0.05) with Mayo scores
